# The anti-ErbB2 antibody H2-18 and the pan-PI3K inhibitor GDC-0941 effectively inhibit trastuzumab-resistant ErbB2-overexpressing breast cancer

**DOI:** 10.18632/oncotarget.17907

**Published:** 2017-05-16

**Authors:** Lingfei Wang, Xiaojie Yu, Chao Wang, Shujun Pan, Beibei Liang, Yajun Zhang, Xiaodan Chong, Yanchun Meng, Jian Dong, Yirong Zhao, Yang Yang, Huajing Wang, Jie Gao, Huafeng Wei, Jian Zhao, Hao Wang, Chaohua Hu, Wenze Xiao, Bohua Li

**Affiliations:** ^1^ Shanghai Key Laboratory for Molecular Imaging, Shanghai University of Medicine and Health Sciences, Shanghai 201318, China; ^2^ International Joint Cancer Institute, Second Military Medical University, Shanghai 200433, China; ^3^ Hangzhou Sanatorium of People's Liberation Army, Hangzhou 310007, China; ^4^ Department of Medical Oncology, Fudan University Shanghai Cancer Center, Shanghai Medical College of Fudan University, Shanghai 200032, China; ^5^ Department of Vascular Surgery, Changhai Hospital, Second Military Medical University, Shanghai, 200433, China; ^6^ Department of Pharmaceutical Sciences, Second Military Medical University, Shanghai 200433, China; ^7^ Department of General Surgery, Xiaogan Central Hospital Affiliated to Wuhan University of Science and Technology, Wuhan 432000, China; ^8^ Department of Rheumatology, Shanghai Pudong Hospital, Fudan University Pudong Medical Center, Shanghai 201399, China

**Keywords:** trastuzumab resistance, GDC-0941, anti-ErbB2 antibody, programmed cell death, breast cancer

## Abstract

Trastuzumab, an anti-ErbB2 humanized antibody, brings benefit to patients with ErbB2-amplified metastatic breast cancers. However, the resistance to trastuzumab is common. Our previously reported H2-18, an anti-ErbB2 antibody, potently induced programmed cell death in trastuzumab-resistant breast cancer cells. Here, we aim to investigate the antitumor efficacy of H2-18 in combination with the pan-PI3K inhibitor GDC-0941 in trastuzumab-resistant breast cancer cell lines. The results showed that H2-18 and GDC-0941 synergistically inhibited the *in vitro* proliferation of BT-474, SKBR-3, HCC-1954 and HCC-1419 breast cancer cells. H2-18 plus GDC-0941 showed significantly enhanced programmed cell death-inducing activity compared with each drug used alone. The combination of H2-18 and GDC-0941 did not increase the effect of single agent on ROS production, cell cycle and ErbB2 signaling. Importantly, the *in vivo* antitumor efficacy of H2-18 plus GDC-0941 was superior to that of single agent. Thus, the enhanced *in vivo* antitumor efficacy of H2-18 plus GDC-0941 may mainly be attributable to its increased programmed cell death-inducing activity. Collectively, H2-18 plus GDC-0941 could effectively inhibit tumor growth, suggesting the potential to be translated into clinic as an efficient strategy for ErbB2-overexpressing breast cancers.

## INTRODUCTION

Breast cancer has been the most commonly diagnosed cancer and the principle cause of cancer-related mortality in women worldwide [1]. It can be classified to ER^+^, ErbB2^+^, and ER^−^PR^−^ ErbB2^−^ groups based on the expression levels of estrogen receptors, progesterone receptors and ErbB2 [1]. According to breast cancer treatment (PDQ^®^), standard treatments of breast cancer can be divided to 5 types: surgery, radiation therapy, chemotherapy, hormone therapy, and targeted therapy. ErbB2-targeted therapy is a successful example as personalized cancer treatment that has made a significant progress in clinical outcomes.

ErbB2 (also known as HER2) is a member of epidermal growth factor receptor family [2]. Overexpression of ErbB2 is found in about 25%–30% of human breast cancers, and is associated with tumorigenesis, cancer progression and poor prognosis [3, 4]. ErbB2 activation is dependent on ErbB2 homodimers or heterodimers with other ErbB family members (ErbB1, 3, 4), which could stimulate constitutive phosphorylation of ErbB2 and initiate the main downstream PI3K/AKT pathway and MAPK pathway, culminating in tumor growth.

Trastuzumab (Herceptin) is the first anti-ErbB2 humanized monoclonal antibody approved by FDA for clinical use for ErbB2-ampified metastatic breast cancers in 1998 [5]. Although it has substantially improved outcomes for patients with ErbB2-positive breast cancer, about 70% of ErbB2-amplified breast cancers do not respond to trastuzumab [6, 7]. Thus, there is an urgent need to develop new strategies to circumvent trastuzumab resistance.

Recent researches have focused on the abnormalities involved in the mechanisms of trastuzumab resistance. Hyperactivated PI3K pathway is such an important abnormality, which exists in over 70% of breast cancers [8, 9]. Aberrant activation of PI3K/AKT signaling pathway could be caused by several growth factor receptors such as IGF1R, HER1 (EGFR) [10, 11] and HER3 [12], or alterations including low PTEN levels and PIK3CA-activating mutations [9, 13–16]. Nevertheless, the human breast cancer cell lines which harbor these aberrant changes in PI3K signaling still remain sensitive to a selective and potent class I PI3K inhibitor GDC-0941. GDC-0941 could inhibit sustained PI3K/AKT signaling pathway and help overcome trastuzumab resistance.

Our previous study has shown that an anti-ErbB2 antibody H2-18 exhibits a unique ability to overcome trastuzumab resistance [17]. It shows a superior antitumor efficacy over trastuzumab plus pertuzumab in trastuzumab-resistant breast cancer cell lines through inducing potent programmed cell death [17]. Due to the heterogeneous nature of breast cancers, one single drug is usually not enough to overcome trastuzumab resistance. Here, we investigated the antitumor effect of H2-18 in combination with the pan-PI3K inhibitor GDC-0941 on both trastuzumab-sensitive (BT-474, SKBR-3) and -resistant (HCC-1954, HCC-1419) breast cancer cell lines.

## RESULTS

### H2-18 and GDC-0941 synergistically inhibit the *in vitro* growth of breast cancer cell lines

**Figure 1:**
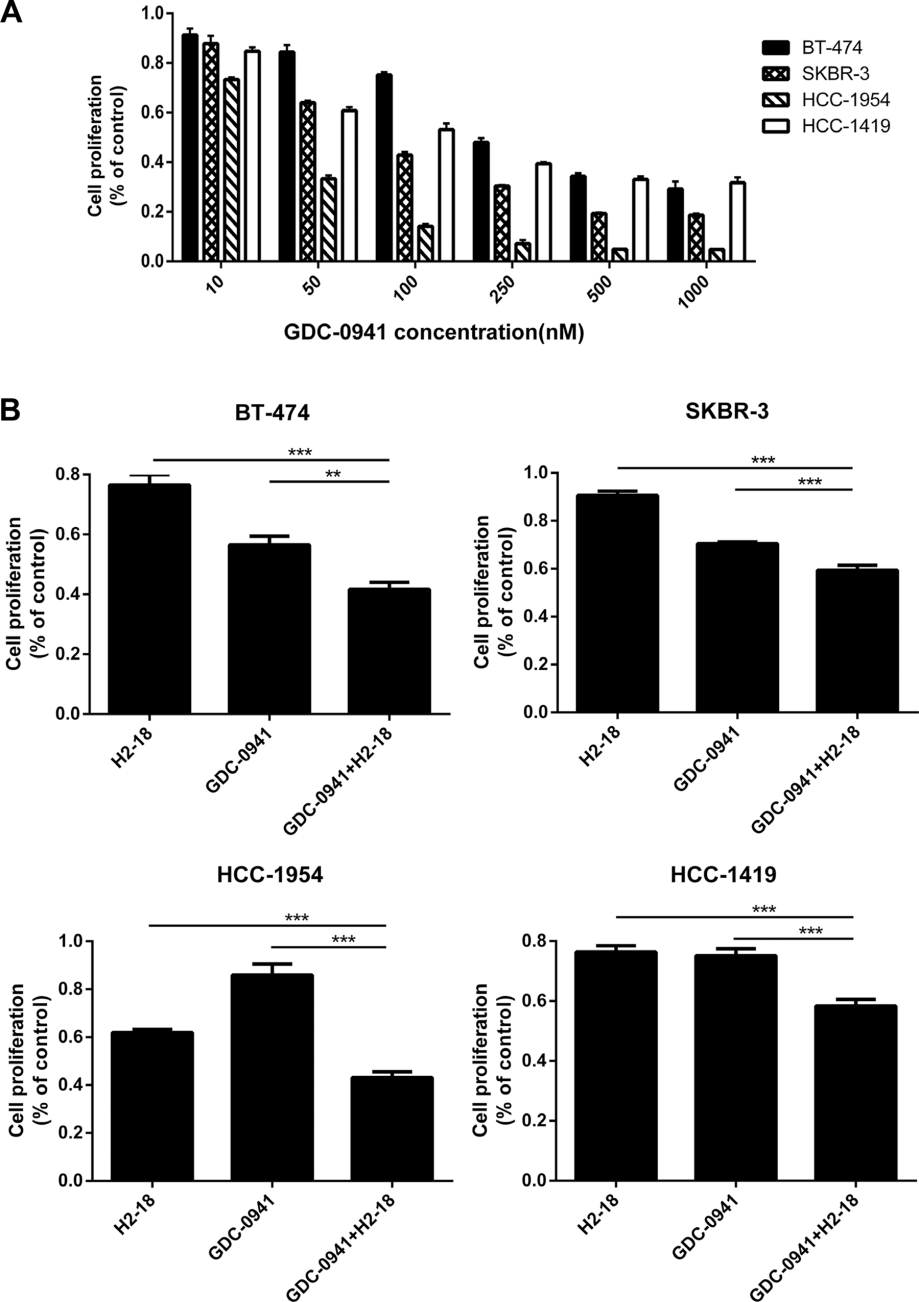
GDC-0941 and H2-18, either used alone or in combination, could effectively inhibit the cell proliferation of breast cancer cell lines SKBR-3, BT-474, HCC-1419 and HCC-1954 (**A**), The IC50 of GDC-0941 in these four cell lines was determined by CCK8 assay. The experiment was performed at least three times at different times. (**B**), CCK8 assay comparing the effects of control IgG, H2-18, GDC-0941, and H2-18 plus GDC-0941 on cell proliferation of breast cancer cell lines SKBR-3, BT-474, HCC-1419 and HCC-1954. Results are shown as percentage of control cell proliferation. Error bars, SD *P < 0.05; **P < 0.01; ***P < 0.001.

We evaluated the ability of GDC-0941 to inhibit the growth of BT-474, SKBR-3, HCC-1954 and HCC-1419 breast cancer cell lines. The results showed that GDC-0941 suppressed the *in vitro* proliferation of both trastuzumab-sensitive (BT-474, SKBR-3) and trastuzumab-resistant (HCC-1954, HCC-1419) cell lines in a dose-dependent manner (Figure 1A). Compared with BT-474, SKBR-3 and HCC-1419 cell lines, HCC-1954 were more sensitive to GDC-0941 (Figure 1A). Next, we evaluated the ability of GDC-0941 and H2-18, either alone or in combination, to inhibit the proliferation of BT-474, SKBR-3, HCC-1954 and HCC-1419 cells. In all these cell lines, GDC-0941 plus H2-18 showed a significantly greater anti-proliferative activity than either agent alone (Figure 1B).

**Figure 2:**
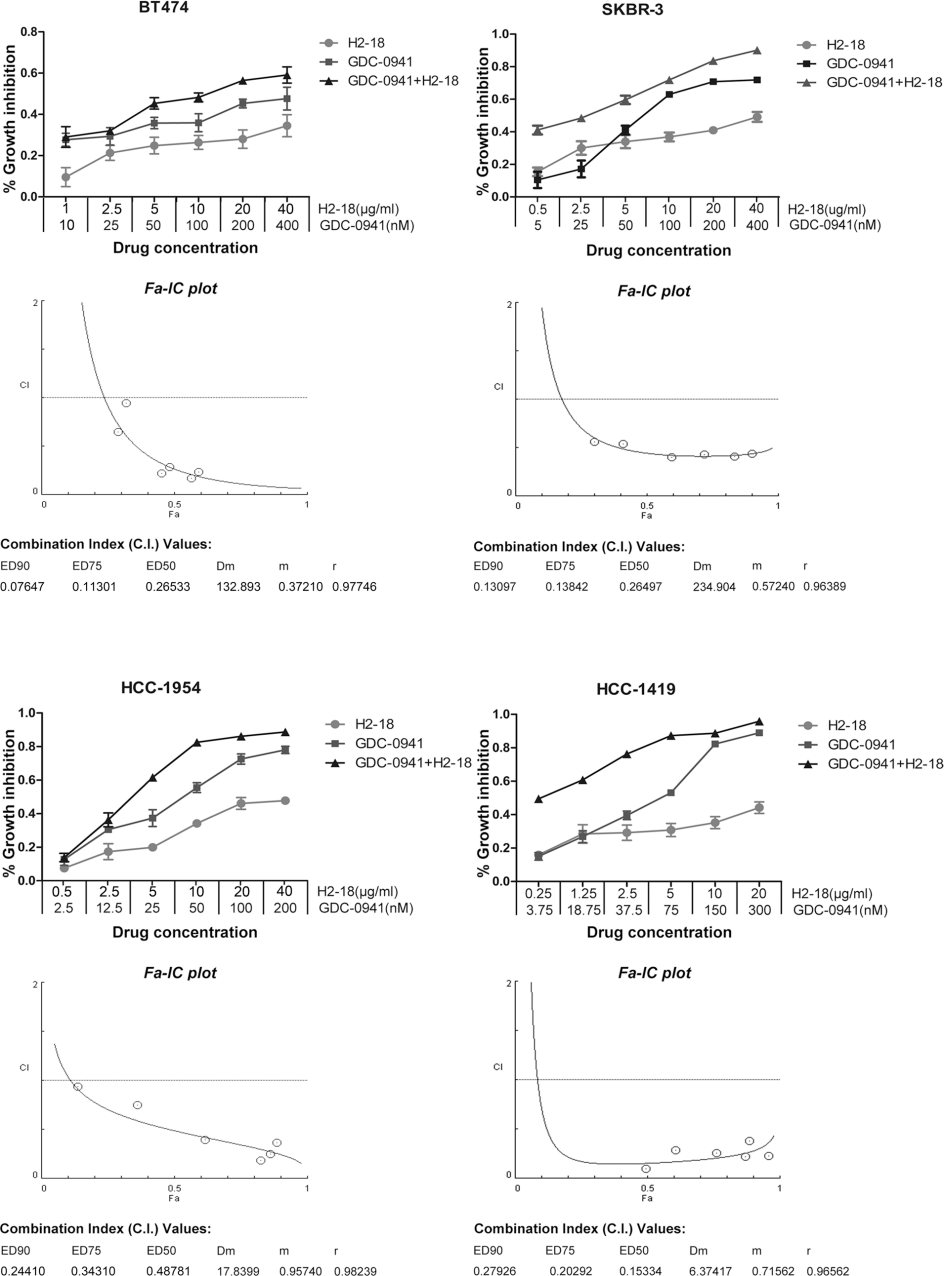
H2-18 and GDC-0941 synergistically inhibited the growth of both trastuzumab-sensitive and -resistant breast cancer cell lines CCK8 assay was used to compare cell proliferation of the breast cancer cell lines SKBR-3, BT-474, HCC-1419 and HCC-1954 upon indicated treatments. Combination index (CI) values were calculated using the Chou-Talalay method. Drug synergy, addition, and antagonism are defined by C.I. values less than 1.0, equal to 1.0, or greater than 1.0, respectively.

Extensive studies of mammary cells including breast cancer cells have revealed that 3D cell culture models could more accurately mimic the *in vivo* signaling, behavior and reaction of cancer cells to drugs than conventional 2D models [17–20]. Here, to further investigate whether the combination of H2-18 and GDC-0941 is synergistic in inhibiting cell proliferation, we treated BT-474, SKBR-3, HCC-1954 and HCC-1419 cells with various concentration ranges of GDC-0941 and H2-18 in 3D culture system. Data were analyzed using the method of Chou and Talalay to establish drug C.I. values. Synergy is defined as C.I. values of < 1.0, antagonism as C.I. values > 1.0, and additivity as CI values equal to 1.0. Our results showed that in both trastuzumab-sensitive and trastuzumab-resistant cell lines, H2-18 and GDC-0941 synergistically inhibited cell proliferation (Figure 2).

### H2-18 plus GDC-0941 inhibits the ErbB2 signaling in breast cancer cell lines

**Figure 3:**
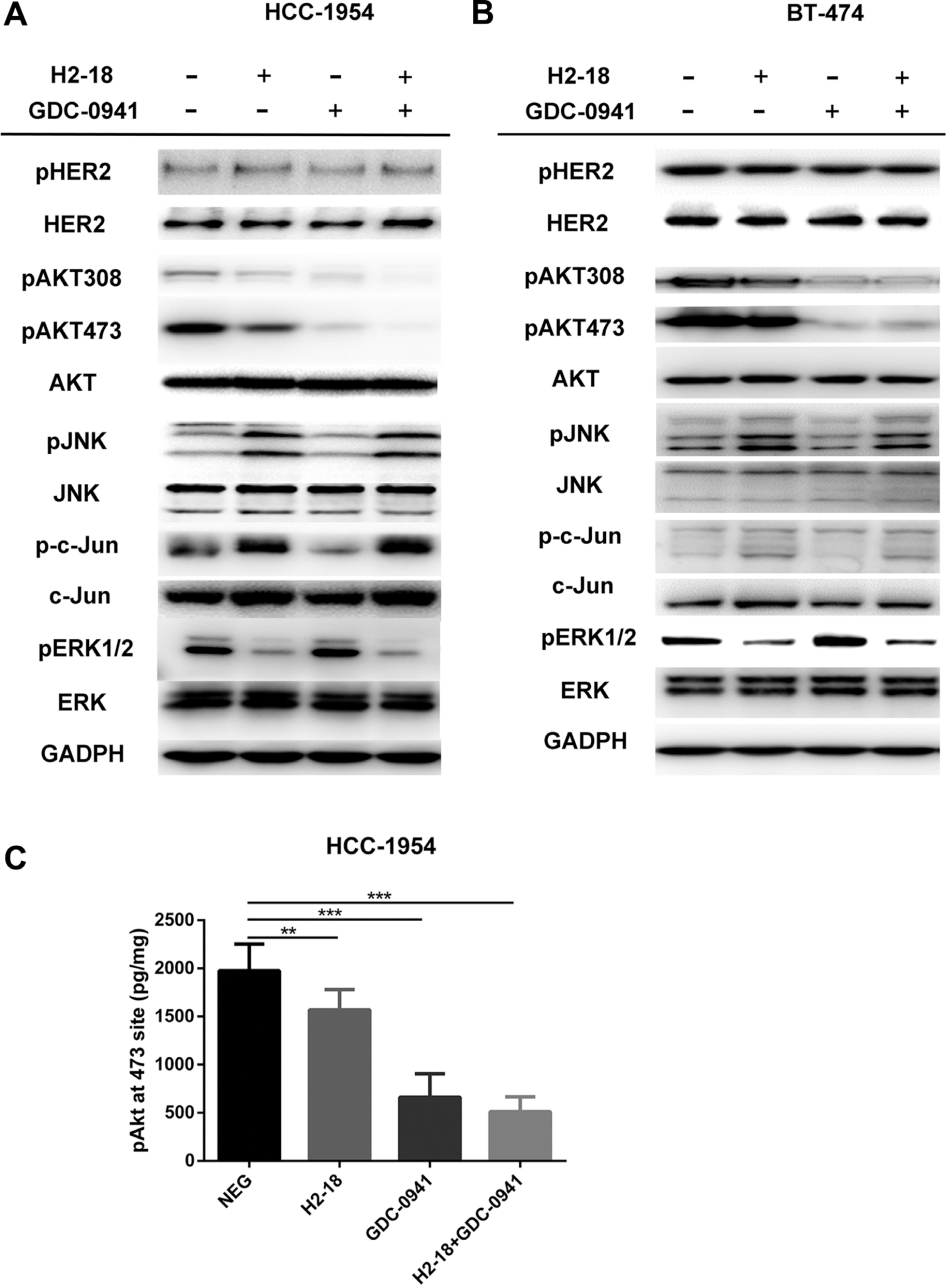
H2-18 plus GDC-0941 inhibits the ErbB2 signaling in breast cancer cell lines HCC-1954 and BT-474 (**A, B**), Immunoblots were used to examine the phosphorylation of ErbB2 signaling in HCC-1954 cells (A) and BT-474 cells (B) treated with control IgG, H2-18, GDC-0941, and GDC-0941 plus H2-18 for 4 h. (**C**), Quantification of Akt phosphorylation in HCC-1954 cells following indicated drug treatments. HCC-1954 cells treated with control IgG, H2-18, GDC-0941, and GDC-0941 plus H2-18 for 4h were lysed immediately, and the concentrations of phospho-Akt in the cell lysates were determined by a commercially available ELISA kit. Data are normalized to protein content and are shown as mean ± SD (*n* = 8 wells/group). *P < 0.05; **P < 0.01; ***P < 0.001.

To examine the combinatory effect of H2-18 and GDC-0941 on ErbB2 signaling, the trastuzumab-sensitive cell line BT-474 and the trastuzumab-resistant cell line HCC-1954 were treated with indicated treatments and then cell lysates were subjected to western blot. No significant difference was detected in ErbB2 phosphorylation of HCC-1954 cells treated with or without indicated drugs (Figure 3A). Similarly, in BT-474 cells, pErbB2 did not change obviously between control group and drug treatment groups (Figure 3B). In HCC-1954 cells, when H2-18 and GDC-0941 were used in combination, AKT-phosphorylation was nearly abrogated (Figure 3A). In BT474 cells, pAkt in cells treated with H2-18 plus GDC-0941 was similar to that with GDC-0941 alone (Figure 3B). In both cell lines, the addition of GDC-0941 to H2-18 did not further increase pJNK or p-c-Jun, and did not further decrease pErk1/2 (Figure 3A, 3B).

Consistently, the results from Elisa showed that GDC-0941 alone could decrease pAkt effectively (Figure 3C). The addition of H2-18 to GDC-0941 could augment its inhibitory effect on pAkt (Figure 3C). However, no significant difference was obtained in HCC-1954 cells between GDC-0941 treatment and H2-18 plus GDC-0941 treatment (Figure 3C).

### The addition of GDC-0941 to H2-18 does not increase ROS production

**Figure 4:**
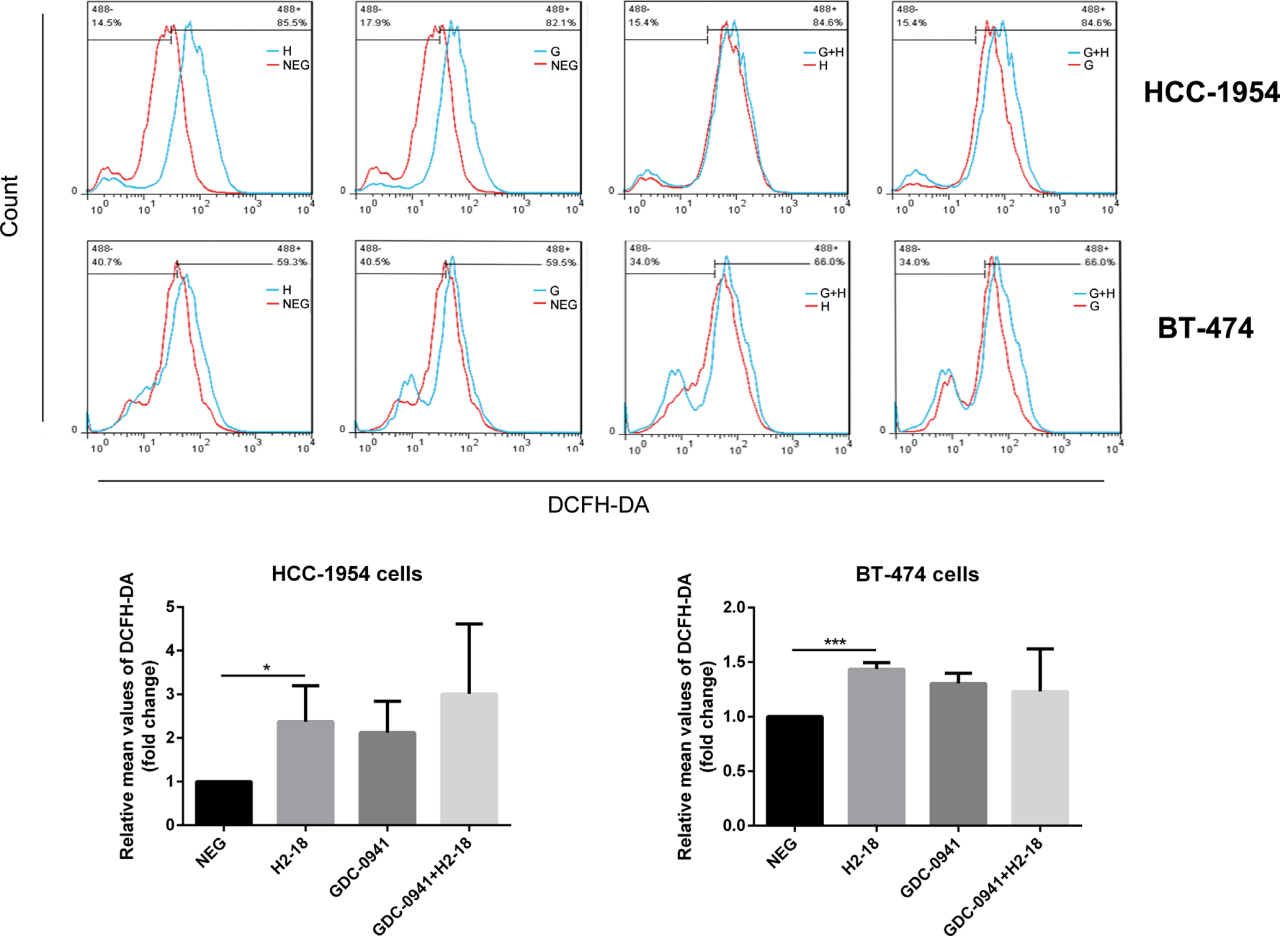
H2-18 plus GDC-0941 did not significantly increase ROS production compared with H2-18 alone DCFH-DA was detected by flow cytometry to measure the level of ROS production in HCC-1954 cells or BT-474 cells treated with control IgG (NEG), H2-18 (H), GDC-0941 (G) and GDC-0941 plus H2-18(G+H). Data from three independent ROS detection experiments at different time were analyzed. *P < 0.05; **P < 0.01; ***P < 0.001; Student unpaired *t* test.

As ROS was involved in programmed cell death induced by H2-18, we explored whether the addition of GDC-0941 to H2-18 would affect ROS production. The results showed that although H2-18 alone could increase the ROS level in HCC-1954 cells. The combination of GDC-0941 and H2-18 exhibited a similar ROS-inducing ability as H2-18 alone (Figure 4). Similar results were also obtained with BT-474 cells (Figure 4).

### H2-18 plus GDC-0941 induces more programmed cell death than agents alone

**Figure 5:**
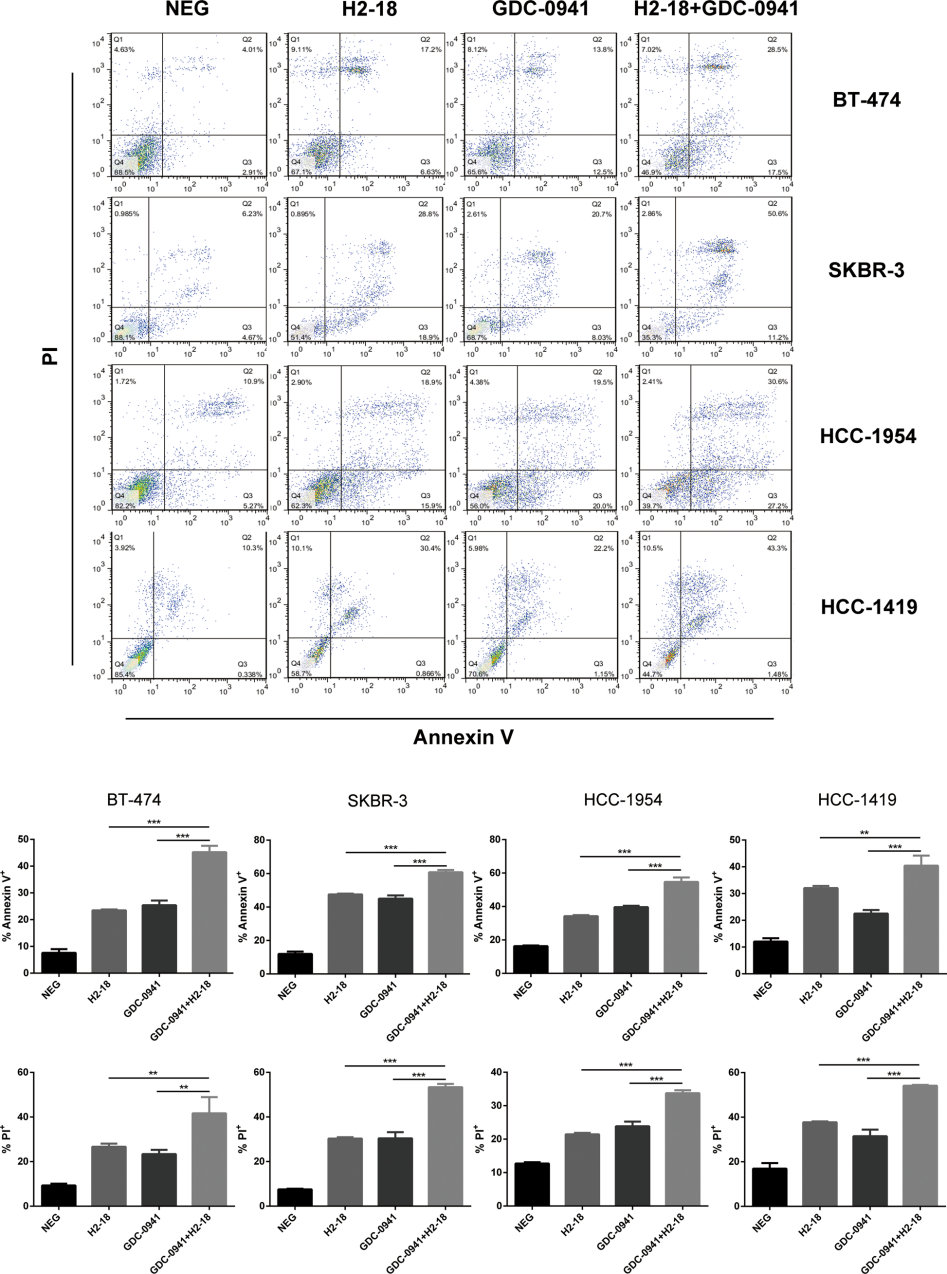
H2-18 and GDC-0941 in combination induced more cell death than agents alone Cell death induced by control IgG, H2-18, GDC-0941, and GDC-0941 plus H2-18 in breast cancer cell lines SKBR-3, BT-474, HCC-1419 and HCC-1954 was examined by flow cytometry using Annexin V/PI double staining detecting kit. Error bars, SD *P < 0.05; **P < 0.01; ***P < 0.001.

Flow cytometry was used to determine the programmed cell death (PCD)-inducing activity of H2-18 and GDC-0941 in BT-474, SKBR-3, HCC-1954 and HCC-1419 cell lines by using FITC Annexin V Apoptosis Detection Kit I. The results showed that in these four cell lines, all of the treatments, H2-18, GDC-0941, and H2-18 plus GDC-0941, could effectively increase cell death (Figure 5). In HCC-1954 cells treated with H2-18 plus GDC-0941, the percentage of Annexin V positive cells is 54.7%, far higher than that of HCC-1954 cells treated with either H2-18 or GDC-0941 alone (Figure 5). The combinatory treatment of H2-18 and GDC-0941 could also induce much more PI positive HCC-1954 cells than did single agent (Figure 5). Similar results were observed with BT-474, SKBR-3, and HCC-1419 cell lines (Figure 5).

### H2-18 plus GDC-0941 alters the cell cycle distribution

Many researches have reported that PI3K activity is involved in cell cycle progression [22]. Next, we assessed cell cycle distribution in HCC-1954 and BT-474 cells after 5 days of drug treatments. In BT-474 cells, H2-18 plus GDC-0941 could induce a G1-phase cell cycle arrest more effectively than either H2-18 or GDC-0941 alone (Supplementary Figure 1). However, in HCC-1954 cells, H2-18 plus GDC-0941 did not display a capability to induce a G1-phase arrest (Supplementary Figure 1).

### H2-18 plus GDC-0941 suppresses the growth of trastuzumab-resistant breast cancer xenografts

**Figure 6:**
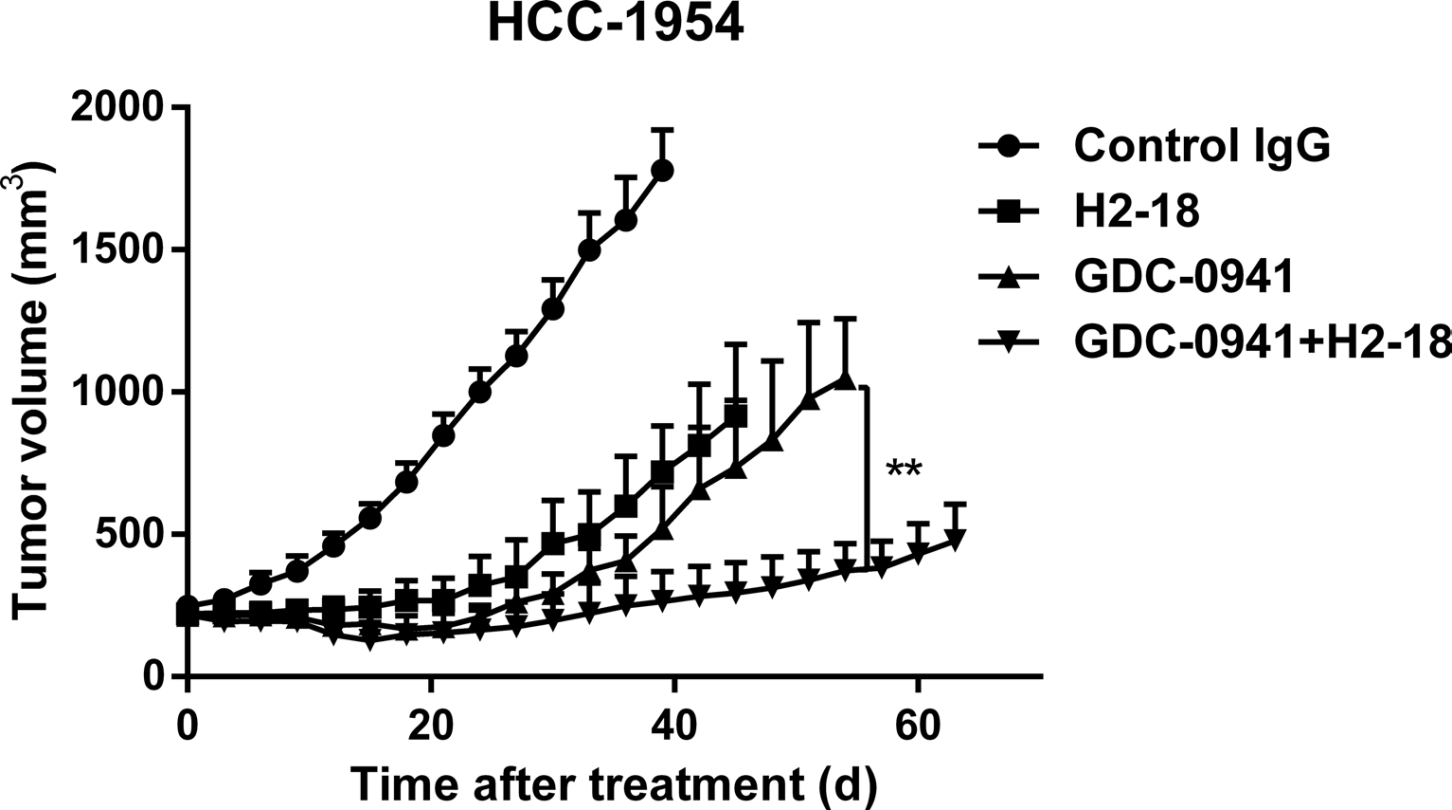
H2-18 plus GDC-0941 suppressed the *in vivo* growth of HCC-1954 xenografts Tumor volume of HCC-1954 breast tumor xenografts were calculated after treatment with control IgG (10 mg/kg, twice a week, intravenously), H2-18 (10 mg/kg, twice a week, intravenously), GDC-0941 (100 mg/kg, daily, orally), and GDC-0941 (100 mg/kg, daily, orally) plus H2-18 (10 mg/kg, twice a week, intravenously). Data are shown as means ± SEM. *P < 0.05; **P < 0.01 Mann-Whitney test.

The therapeutic efficacy of H2-18, GDC-0941, and H2-18 plus GDC-0941 was examined in nude mice bearing established HCC-1954 xenograft tumors. H2-18 and GDC-0941 were almost equally effective at inhibiting the HCC-1954 tumor growth (Figure 6). Importantly, the combination of H2-18 and GDC-0941 exhibited a much greater inhibitory effect on HCC-1954 xenograft growth than either of these two drugs used alone (Figure 6).

## DISCUSSION

An increasing number of researches attempt to elucidate the mechanisms of trastuzumab resistance. Genetic alterations in ErbB2 or its downstream signaling targets were one such important mechanism. The latter alterations mainly include PIK3CA-activating mutations and phosphatase and tensin homolog (PTEN) deficiency. They lead to aberrantly sustained activation of PI3K/AKT pathway [14, 23–26], which exists in over 70% of breast cancers [8, 9] and could be inhibited by PI3K inhibitors. Compared with other kinds of PI3K inhibitors, GDC-0941, an orally bioavailable inhibitor of class I PI3K, displays more favorable pharmacokinetic and toxicological properties [27] and is now in clinical development for many tumors including breast cancer [27–29]. GDC-0941 inhibits class I PI3K activity directly and potently, suppressing aberrant activation of PI3K/AKT pathway. GDC-0941 was also reported to arrest cells in G1-phase and induce apoptosis [30]. Thus, even though breast cancer cell lines harbor PIK3CA mutations or PTEN deficiency, such as HCC-1954 and HCC-1419, they are still sensitive to GDC-0941.

In our recent study, we reported an ErbB2 domain I-specific fully human antibody H2-18, which exhibited a great antitumor activity in trastuzumab-resistant breast tumor-bearing nude mice [17]. However, H2-18 only had a slight inhibition effect on PI3K/AKT pathway. Further studies suggested that the *in vivo* antitumor efficacy of H2-18 may be mainly attributable to its potent PCD-inducing activity. Programmed cell death is a cell suicide event, playing a key role in both normal development and pathogenesis [31–33]. PCD including programmed necrosis and apoptosis is executed and regulated by delicate mechanisms [34]. Even when cancer cells are resistant to PI3K blockade, PCD could still act as an effective mechanism to accelerate cell death [35]. In this study, we asked if the combination of a potent inhibitor of PI3K/AKT pathway and the potent PCD-inducing anti-ErbB2 antibody H2-18 may achieve a greater antitumor efficacy. Our results indicated that GDC-0941 plus H2-18 could inhibit the growth of trastuzumab-resistant breast cancer cells more effectively than either GDC-0941 or H2-18 alone, both *in vitro* and *in vivo*. More importantly, cell death induced by H2-18 was substantially increased by the addition of GDC-0941. Additionally, the combination of GDC-0941 and H2-18 did not exert a more potent effect on inhibition of Akt phosphorylation, induction of G1-phase cell cycle arrest and increase in ROS production than the single agent. Thus, the superior antitumor activity of H2-18 plus GDC-0941 may be mainly attributable to its increased PCD-inducing capacity.

Compared with other studied breast cancer cell lines, HCC-1954 seems to be more sensitive to H2-18 plus GDC-0941. We think the reason may be as follows: 1, The PCD-inducing ability of H2-18 plus GDC-0941 is similar in different cell lines.2, Different breast cancer phenotypes may exert different drug response. H2-18 exhibited a more potent cell proliferation inhibitory activity in HCC-1954 than other cell lines. In our study, GDC-0941 is also more effective in cell proliferation inhibition of HCC-1954 cells. H2-18 plus GDC-0941 showed a more potent ability to inhibit cell proliferation of HCC-1954 than that of other cell lines. 3, Our results showed that H2-18 could inhibit MAPK pathway. Moreover, H2-18 is more effective, albeit slightly, in inhibiting pAkt in HCC-1954 cell line than in BT-474 cell line. Thus, in HCC-1954 cells, H2-18 plus GDC-0941 nearly completely block the important downstream signal pathways of ErbB2: the MAPK and the PI3K pathways. The exact mechanisms underlying the sensitivity of HCC-1954 cells to the combination of drugs need to be studied in our further research.

In conclusion, due to the heterogeneous nature of tumors, targeting one molecule or one resistance mechanism is usually inefficient [36]. Here, H2-18 plus GDC-0941 was significantly more effective than either agent alone in inhibiting the growth of breast cancer cell lines, suggesting that the combination of drugs with complementary mechanisms could achieve a greater antitumor activity in trastuzumab-resistant breast cancer cell lines. This is the first study to investigate the synergetic antitumor activity of the combination of a PI3K inhibitor and an anti-ErbB2 antibody with potent PCD-inducing activity. Our study also suggested that the combination of H2-18 with GDC-0941 may be a promising effective strategy for the treatment of ErbB2-overexpressing breast cancer.

## MATERIALS AND METHODS

### Cell lines and animals

The ErbB2-overexpressing human breast cancer cell lines SKBR-3, BT-474, HCC-1954 and HCC-1419 were purchased from the American Type Culture Collection (ATCC, Manassas, VA) and grown in either DMEM:F12 (SKBR-3) or RPMI-1640 (HCC-1419, HCC-1954, BT-474) supplemented with 10% fetal bovine serum. All the cell lines were authenticated twice by morphologic and isoenzyme analyses during the study period. Cell lines were routinely checked for contamination by mycoplasma using Hoechst staining and consistently found to be negative. Six-week-old female BALB/c nude mice were obtained from the Shanghai Experimental Animal Center of Chinese Academy of Sciences (Shanghai, China). All the animals were treated in accordance with guidelines of the Committee on Animals of the Second Military Medical University.

### Three-dimensional cell culture

Flat-bottomed 96-well plates were coated with a thin layer of Matrigel (BD Biosciences) on ice and then placed in the 37°C incubator for approximately 30 min to allow for matrix polymerization. Cells were digested with trypsin, resuspended in their respective growth media and added to the precoated plates. After about 30 min incubation, control IgG, H2-18, GDC-0941, or H2-18 plus GDC-0941 in cell culture medium supplemented with 10% Matrigel was added onto the plates gently. Cell culture media were refreshed every 2 to 3 d for a total duration of 5 d.

### Dose-response studies

The pan-PI3K inhibitor GDC-0941 was purchased from Selleck Ltd. (Shanghai, China). Cells were seeded at a density of 5000 cells per well in a flat-bottomed 96-well plate at 37°C in a humidified and 5% CO2 atmosphere. Next day after seeding, cells were incubated with an increasing concentration of GDC-0941. The medium was replaced every 2 or 3 days. Five days later, cell proliferation was determined by Cell Counting Kit CCK-8/WST-8 (DOJINDO, Japan).

### Analysis of combined drug effects

Three-dimensional culture models of breast cancer were utilized to investigate the effect of indicated drugs as single or in combination. After cells in 3D cell culture system were treated with indicated drugs for 5d as mentioned above, the cell viability was determined by CCK8 assay. Percentage of cell growth inhibition was calculated as [1-(treated cells /untreated cells) × 100%]. Combination index (CI) values were calculated using CompuSynsoware program (ComboSyn Inc., Paramus, NJ, USA) by the Chou-Talalay method. C.I. values < 1.0 indicates drug synergy, C.I. values = 1.0 represents drug addition, and C.I. values > 1.0 means drug antagonism.

### Immunoblot analysis

HCC-1954 cells were treated with control IgG (30 μg/ml), H2-18 (30 μg/ml), GDC-0941 (80 nM), and H2-18 (30 μg/ml) plus GDC-0941 (80 nM) for 6 h at 37°C. And BT-474 cells were treated with control IgG (30 μg/ml), H2-18 (30 μg/ml), GDC-0941 (400 nM), and H2-18 (30 μg/ml) plus GDC-0941 (400 nM) for 6 h at 37°C. After washing, cells were lysed in SDS lysis buffer supplemented with protease inhibitors cocktail (Selleck, China). Cell lysates were subjected to SDS-PAGE, transferred to polyvinylidene difluoride (PVDF) membranes and immunoblotted with monospecific antibodies against ErbB2 p-ErbB2(Tyr1221/1222), AKT, p-AKT(S473), p-AKT(T308), p44/42 MAPK, phospho-p44/42 MAPK(T202/Y204), SRC, p-SRC(Y416), p-SRC(Y527), SAPK/JNK, p-SAPK/JNK(T183/Y185), c-Jun, p-c-Jun(S63). After washing, PVDF was incubated with a Horseradish peroxidase-conjugated secondary antibody. Finally, detection was performed using the enhanced chemiluminescence reagents (Plus-ECL) (PerkinElmer, MA, USA).

### Measurement of phorspho-Akt by ELISA

HCC-1954 cells were seeded in a flat-bottomed 96-well plate in humidified 37°C and 5% CO2 atmosphere. After a 24 h-attachment, the cells were treated with control IgG (30 μg/ml), H2-18 (30 μg/ml), GDC-0941 (80 nM), and H2-18 (30 μg/ml) plus GDC-0941 (80 nM) for 6 h. Then cells were lysed according to the manufacturer's instructions (Duoset IC ELISA kit for phospho-Akt, R&D system) and then added into a 96-well microplate precoated with phospho-Akt capture antibodies. After 2 h-incubation, the microplate was washed with wash buffer and incubated with p-Akt (S473) detection antibodies for 1h. Finally, the relative amount of phospho-Akt protein was detected using a standard streptavidin-HRP format and the absorbance was read at 450 nm. The value of phospho-Akt was normalized to the protein content of each well before statistical analysis.

### Cell death assay

Cells were seeded in flat-bottomed 24-well plate at a density of 1 × 10^5^ cells per well in the growth medium and grown in a 37°C humidified incubator with 5% CO_2_. Next day, cells were treated with control IgG, H2-18, GDC-0941, and H2-18 plus GDC-0941 for 24 h. The concentration of GDC-0941 used was decided according to different cell lines. Cell death was measured according to the manufacturer's protocol (FITC Annexin V Apoptosis Detection Kit I, BD Biosciences). Briefly, cells were harvested, washed with binding buffer and then resuspended in 100 μl binding buffer. Five μl FITC-conjugated Annexin V and 5 μl PI were added to the cell suspension and incubated for 15 min in dark at room temperature. Then, the cells were washed with binding buffer, resuspended in 200 μl binding buffer, and finally analyzed by flow cytometry on a FACSCalibur (Becton Dickinson).

### ROS detection

2′, 7′-dichlorofluorescin diacetate (DCFH-DA, Sigma) was used to assess ROS level. Cells were seeded at a density of 1 × 10^5^ cells per well in a flat-bottomed 24-well plate. After cells were treated with control IgG, H2-18, GDC-0941 and H2-18 plus GDC-0941 for 4 h, cells were collected and incubated with 100μl PBS containing 10μM DCFH-DA for 20 min at 37°C. Extracellular DCFH-DA was then removed by washing the cells twice with PBS. The fluorescence of the cells loaded with DCFH-DA was measured with FACSCalibur (Becton Dickinson).

### Cell cycle analysis

Cells were collected and fixed in 70% ethanol at 4°C overnight to permeabilize the cells. Then, the cells were resuspended in 500 μl stain solution containing 25 μl propidium iodide plus 10 μl RNase A (Beyotime, China) and incubated for 30 min at 37°C. Flow cytometric analyses were carried out in a fluorescence-activated sorter (FACSCalibur, Becton Dickinson). Analysis of cell cycle was performed by the software Modfit Lt 3.0 (Verity Software House, USA).

### Mouse xenograft studies

HCC-1954 cells (5 × 10^6^ per mouse) were inoculated subcutaneously into the right flank of 6 weeks old female BALB/c nude mice. When tumor volumes reached an average of about 200 mm^3^, the mice were randomly divided into groups of 10 mice each. Mice were intravenously injected with control human IgG (10 mg/kg) or H2-18 (10 mg/kg) twice weekly. GDC-0941 was prepared in 0.25% (w/v) sodium carboxymethyl cellulose (CMC-Na) suspension. We treated mice with control (0.25% CMC-Na), or GDC-0941 (100 mg/kg) daily via oral gavage. Tumors were measured with digital calipers, and tumor volumes were calculated by the formula: volume = [length × (width) ^2^] / 2. No significant animal weight loss and toxicities were observed in the *in vivo* experiments.

### Statistical analysis

Statistical analysis was performed by ANOVA test to identify significant differences unless otherwise indicated. Differences were considered significant at *P <* 0.05.

## SUPPLEMENTARY FIGURE


