# Supplementary material for: The anti-ErbB2 antibody H2-18 and the pan-PI3K inhibitor GDC-0941 effectively inhibit trastuzumab-resistant ErbB2-overexpressing breast cancer
Source: Oncotarget. 2017 May 16;8(32):52877–88. doi: 10.18632/oncotarget.17907 (PMC5581078; doi:10.18632/oncotarget.17907)
Supplement: Supplementary file 1 [file oncotarget-08-52877-s001.pdf]

The anti-ErbB2 antibody H2-18 and the pan-PI3K inhibitor GDC-0941 effectively inhibit trastuzumab-resistant ErbB2-overexpressing breast cancer

SUPPLEMENTARY MATERIALS

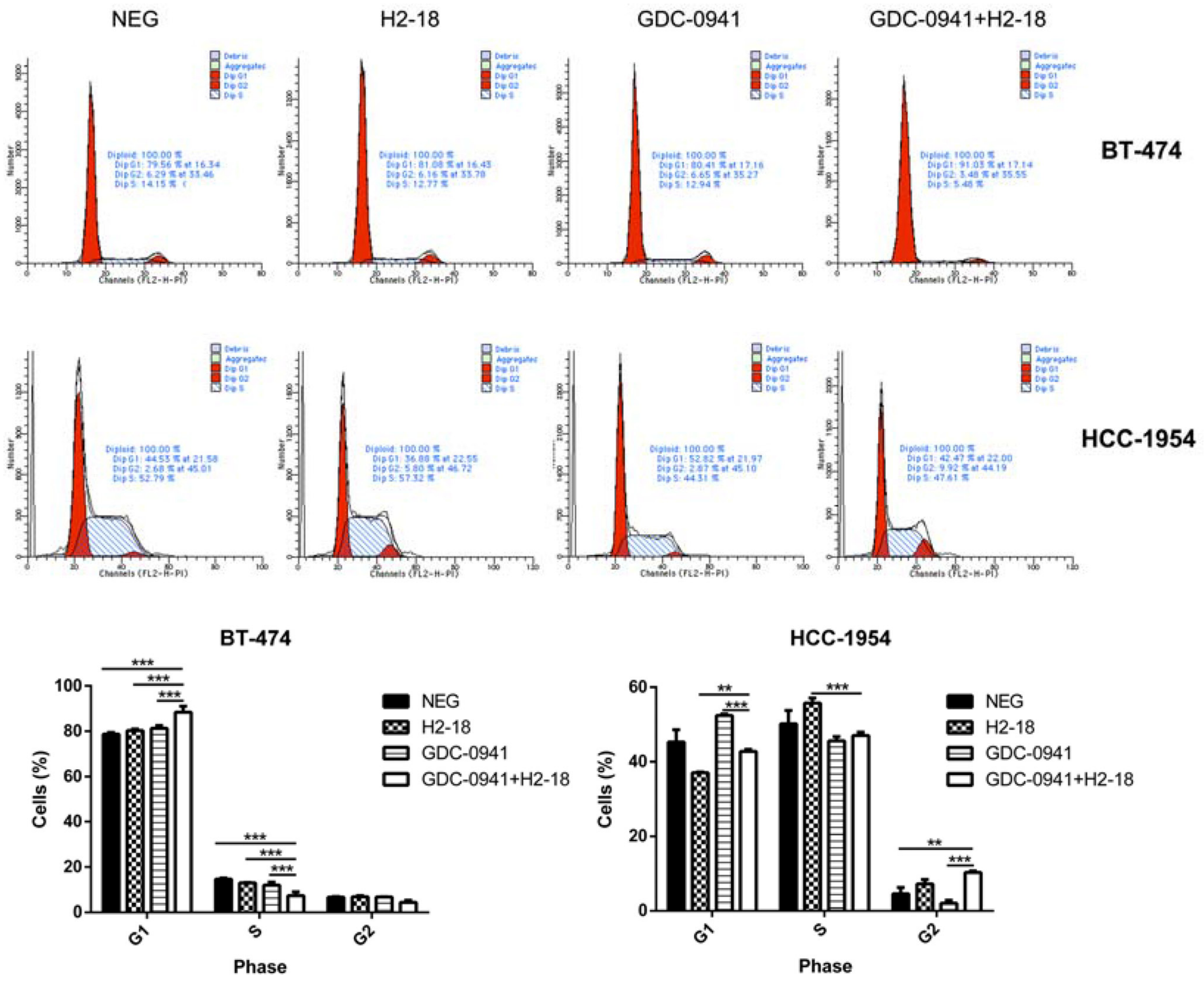

Supplementary Figure 1: The combination of H2-18 and GDC-0941 influenced the cell cycle distribution. HCC-1954 cells and BT474 cells were exposed to control IgG, H2-18, GDC-0941, and GDC-0941 plus H2-18 for 5 days. Subsequently, the cell cycle distribution was determined by PI staining. Statistically significant differences are marked by asterisk (*$P < 0.05$;**$P < .01$;***$P < 0.001$). The data shown here represents three independent experiments.
